# Machine Learning-Based View Synthesis in Fourier Lightfield Microscopy

**DOI:** 10.3390/s22093487

**Published:** 2022-05-03

**Authors:** Julen Rostan, Nicolo Incardona, Emilio Sanchez-Ortiga, Manuel Martinez-Corral, Pedro Latorre-Carmona

**Affiliations:** 1 Departamento de Ingenieria Informatica, Universidad de Burgos, E09006 Burgos, Spain; jrs1002@alu.ubu.es (J.R.); plcarmona@ubu.es (P.L.-C.); 2 3D Imaging and Display Laboratory, Department of Optics, University of Valencia, E46100 Burjassot, Spain; emilio.sanchez@uv.es (E.S.-O.); manuel.martinez@uv.es (M.M.-C.); 3 School of Science, Universidad Europea de Valencia, Passeig de l’Albereda, 7, E46010 Valencia, Spain

**Keywords:** Fourier lightfield microscopy, view synthesis, neural radiance fields, 3D microscopy

## Abstract

Current interest in Fourier lightfield microscopy is increasing, due to its ability to acquire 3D images of thick dynamic samples. This technique is based on simultaneously capturing, in a single shot, and with a monocular setup, a number of orthographic perspective views of 3D microscopic samples. An essential feature of Fourier lightfield microscopy is that the number of acquired views is low, due to the trade-off relationship existing between the number of views and their corresponding lateral resolution. Therefore, it is important to have a tool for the generation of a high number of synthesized view images, without compromising their lateral resolution. In this context we investigate here the use of a neural radiance field view synthesis method, originally developed for its use with macroscopic scenes acquired with a moving (or an array of static) digital camera(s), for its application to the images acquired with a Fourier lightfield microscope. The results obtained and presented in this paper are analyzed in terms of lateral resolution and of continuous and realistic parallax. We show that, in terms of these requirements, the proposed technique works efficiently in the case of the epi-illumination microscopy mode.

## 1. Introduction

The realistic generation of different views of an existing scene not only requires understading its three-dimensional (3D) geometry, but also modeling the complex and inherent viewpoint-dependent information resulting from a series of highly non-linear light propagation processes. In these situations, the 5D plenoptic function is a good strategy to model the radiance and direction of the light rays passing through each point in space [1,2,3]. The main difficulty in this approach, however, lies in adequately sampling the plenoptic function.

As a recent alternative to this type of strategy, the novel view synthesis (NVS) framework aims to approximate the plenoptic function from only a sparse group of observations. One possible way of accomplishing this is through the use of a small set of images acquired from different viewpoints. This group of techniques is currently obtaining high-quality results, in particular when being used in a deep learning framework [4]. Their aim is to obtain new views that did not previously exist. Sometimes, other useful scene information might also be subsequently obtained—for instance, the depth maps or the 3D location of the acquisition cameras.

Once the 3D representation is inferred, one might select any new (x,y,z) coordinate to generate a new view from it. Many of these methods allow renders and models of, for instance, people or monuments, therefore enhancing virtual reality (VR) capabilities [5,6].

The neural radiance field (NeRF) method [7] is based on the concept of neural rendering, which aims to substitute some of the rendering stages for a specific type of neural network surrogate. In this framework, once the 3D model is obtained, new views can be synthesized. NeRF aims at using the concept of high-quality real-time ray tracing [8]. The method presented in [7] may be able to represent a non-dynamic scene as a continuous 5D function that outputs the radiance emitted in each direction (θ,ϕ) at each point (x,y,z) in space, and a density parameter at each point which controls how much radiance is accumulated by a ray passing through the 3D (x,y,z) position. Here, θ and ϕ are the zenith and azimuth angles codifying the direction of a unit vector in 3D space.

This method optimizes a multilayer perceptron (MLP) to represent this function by a regression procedure from a single 5D coordinate point (x,y,z,θ,ϕ) to a single volume density and view-dependent RGB color. The original NeRF version makes use of a software called COLMAP [9,10]. COLMAP is a general-purpose structure-from-motion (SfM) and multi-view stereo (MVS) framework (pipeline). It has a varied group of reconstruction methods implemented, allowing for the estimation of the camera system acquisition parameters.

At present, the amount of methods based on (or inspired by) the original NeRF proposal have substantially increased. They have tried to tackle some of the drawbacks of the original theory. Some examples of these new methods include RegNeRF [11], where a series of regularization terms are included to help improve the scene reconstruction quality, InstantNeRF [12], which aimed to increase the convergence speed of the method, pixelNeRF [13], where the main problem to solve is related to the possibility to have one, two, or just a few input images, and D-NeRF [14], which aimed to reconstruct highly dynamic scenes (considering rigid and non-rigid motions), to cite only a few cases.

More recently, Wang et al. [15] presented NeRF--, a more versatile version of the method proposed in [7], which can be applied without any prior knowledge of the acquisition system parameters. This was possible due to the use of a particular type of joint optimization strategy applied during the training process. Some further details about NeRF and NeRF-- will be given in Section 2.

The aim of this paper is to show the utility of NeRF-- in Fourier lightfield microscopy (FLMic) [16,17,18,19,20,21], which is a promising technique for the rapid acquisition of 3D information of microscopic samples. In fact, this system is able to acquire angular and spatial information of the sample in a single shot. This makes FLMic specially suited for the study of dynamic processes in 3D microscopy, such as neural activity [22,23] or single-molecule localization [24]. The lightfield scattered or emitted by the sample can be computationally retrieved through different approaches. The 3D reconstruction of the sample can be computed based on back-propagation algorithms [25], deconvolution-based techniques [26,27], or even deep learning methodologies [28].

There are substantial differences between the conventional perspective images and the ones provided by FLMic. NeRF-- was developed and applied to images that show a conical perspective; that is, the magnification decreases proportionally to the distance. They also all have a positive disparity, which decreases with the distance and is zero for very far-away objects—ideally placed at the infinite. On the contrary, the elemental images captured with FLMic are orthographic, have no disparity (or parallax) at the object plane and, as a consequence, they may have a positive or negative disparity. In this context, we demonstrate the synthesis of elemental images in Fourier lightfield microscopy. This has great utility in the display of 3D microscopic images, since it allows the off-line observation of 3D samples from many different view points with smooth and realistic perspective changes.

In this paper, we demonstrate the applicability of NeRF-- to the microscopic images captured with FLMic. We apply the method to different kinds of samples and with different configurations of the optical system. Then, we discuss the quality of the results, and their dependence on the sample type.

## 2. Materials and Methods

The particular details of the acquisition and processing tools and methodologies are as follows. Section 2.1 is dedicated to describing the main properties of FLMic. Section 2.2 will give a brief summary about the particular NVS method applied on the images acquired by this microscope.

### 2.1. Fourier Lightfield Microscopy

**Figure 1:**
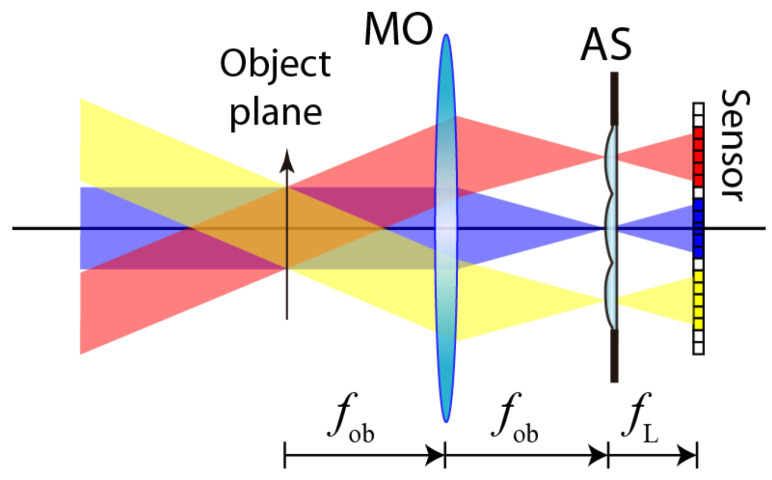
Basic scheme of the Fourier lightfield microscope. The MLA is placed at the aperture stop (AS) of the microscope objective (MO). Note that the object is assumed to be of a 3D nature. Thus, only one section of it (the one placed at the so-called object plane) is conjugated with the sensor.

Lightfield microscopy is an active area of research whose origins can be traced back to the seminal work by Levoy et al. in [29]. FLMic is an upgrade of lightfield microscopy, based on the spatial multiplexing of the angular information of the sample, in such a way that the captured image (named here as the integral image) consists of several elemental images (EIs), each one of them obtained from a different perspective angle. This is achieved by inserting a microlens array (MLA) at the aperture stop (AS) of a telecentric microscope objective (MO). As shown in Figure 1, this telecentricity allows for the acquisition of orthographic EIs, whose lateral magnification does not depend on the object position. Another singular feature is that the object plane is captured with no parallax; i.e., all the EIs are identical for that plane. The number of EIs along one direction, $N_{EI}$, is the result of dividing the AS diameter by the microlenses’ pitch (i.e., the distance between the microlenses’ centers). Plane objects in the space between the object plane and the MO are captured with positive parallax, while the rest are captured with negative parallax. Note that, in conventional perspective setups, the plane with no parallax is placed at the infinite.

One of the main drawbacks of FLMic is that the angular information acquisition is achieved at the cost of reducing the lateral resolution of the EIs. This unavoidable effect occurs due to the spatial multiplexing of the aperture stop, which reduces the effective spatial bandwidth for each EI. In particular, the lateral resolution limit of captured EIs can be expressed as:

(1) $$\rho =\frac{\lambda \;N_{EI}}{2\;\mathrm{NA}},$$

where λ is the wavelength of the light scattered (or emitted in the case of fluorescence) by the sample, $N_{EI}$ is the number of EIs along the corresponding direction, and NA is the numerical aperture of the host microscope objective. Equation (1) shows a loss in lateral resolution (with respect to a conventional microscope) by a factor $N_{EI}$, and, therefore, a direct trade-off between the lateral and the angular resolutions of the system.

Furthermore, the number of EIs is directly related to the 3D information that can be computationally extracted from the sample. For instance, in a set of refocused images (namely, z-stack) assessed with standard back-propagation algorithms, the thickness of the refocused region is inversely proportional to $N_{EI}$. The same tendency occurs when applying deconvolution techniques to provide computational optical sectioning, as the thickness of the computed optical sections decreases (i.e., the optical sectioning capability is improved) when the number of EIs increases [25].

**Figure 2:**
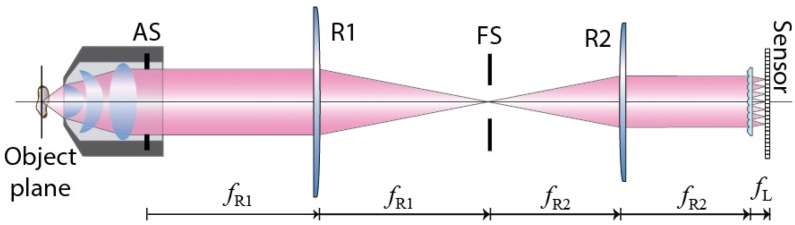
Fourier lightfield microscope optical scheme. The afocal relay R1–R2 projects an image of the aperture stop (AS) onto the lens array. Between them, a field stop (FS) is placed at the plane of the intermediate image, to avoid overlapping between the EIs formed by the lens array. The sensor is placed at the back focal plane of the lens array, so that it conjugated to the object plane.

In a realistic implementation of FLMic, the microlenses are not placed directly at the aperture stop, but in a plane conjugated with it. The standard scheme of FLMic is shown in Figure 2. An afocal relay system (R1–R2) projects an image of the aperture stop onto the lens array. In addition, a field stop (FS) is used to avoid the vignetting and the overlapping between EIs.

### 2.2. Neural Radiance Field, with Simultaneous Inference of Calibration Parameters

The number of EIs plays an important role during both the acquisition and the computational post-processing processes. We must also bear in mind that the 3D information obtained from a scene increases in quality with the increase in the number of EIs processed. That is the reason why NVS methods are attractive in applications that depend on the number of EIs processed.

Given a set of images $I=\{I_{1},\ldots ,I_{N}\}$ captured from *N* sparse viewpoints of a scene, with their associated (intrinsic and extrinsic) camera parameters $\Pi =\{\pi_{1},\ldots ,\pi_{N}\}$, the goal of NVS is to come up with a scene representation that enables the generation of realistic images from novel, unseen viewpoints.

The neural radiance field method [7] represents an ideal tool to optimize the lateral resolution as well as the thickness of the reconstructed axial regions. Nevertheless, as previously stated, NeRF needs a previous calibration step (inference of the intrinsic and extrinsic parameters of the acquisition set-up used). Sometimes, these parameters are difficult or even impossible to obtain. Hence, we considered the use of NeRF--, since it is able to infer the camera parameters during a global optimization process.

The creators of NeRF adopt a continuous function for constructing a volumetric representation of the scene from a sparse group of input views. In essence, it models the view-dependent appearance of the 3D space using a continuous function $F_{\Theta }:\left(x,u\right)\to \left(c,\rho \right)$, parameterized by a multi-layer perceptron (MLP). The function maps a 3D location x=(x,y,z), together with a viewing direction u=(ϑ,ψ), to a radiance color c=(r,g,b) and a density value, ρ. Rendering an image in a NeRF framework implies that the color at each pixel $p=(p_{x},p_{y})$ on the image plane $\left({\hat{I}}_{i}\right)$ is obtained by a so-called rendering function, which aggregates the radiance along a ray coming from the camera position $o_{i}$ and passing through a specific pixel (p) into a volume [3]:

(2) $${\hat{I}}_{i}\left(p\right)=\int_{h_{n}}^{h_{f}}\tau \left(h\right)\rho \left(r\left(h\right)\right)c\left(r\left(h\right),u\right)dh$$

where $\tau \left(h\right)=e^{\left(-\int_{h_{n}}^{h}\rho \left(r\left(s\right)\right)ds\right)}$ denotes the accumulated transmittance along the ray, i.e., the probability of the ray travelling from $h_{n}$ to *h* without hitting any other particle, and r(h)=o+hu denotes the camera ray that starts from camera origin o and passes through p, controlled by the camera parameter $\pi_{i}$, with near and far bounds $h_{n}$ and $h_{f}$. With this implicit scene representation $F_{\Theta }\left(x,u\right)$, NeRF can be trained by minimizing the photometric error between the observed views and the synthesized ones, under known camera parameters: $E=\sum_{i=1}^{N}{∥I_{i}-{\hat{I}}_{i}∥}_{2}^{2}$, where $\Theta^{*}=\arg\min_{\Theta }\left[E\left(\hat{I}|I,\Pi \right)\right]$. $\hat{I}$ denotes the set of synthesized images $\left\{{\hat{I}}_{1},\ldots ,{\hat{I}}_{N}\right\}$. The discretization approach corresponding to Equation (2), and other particular details related to the implementation strategy, can be found in [7].

In contrast to the work in [7], the authors in [15] show that the pre-processing step of estimating the camera parameters $\pi_{i}$ of the input images is unnecessary. Unlike the training setup of the original NeRF, the authors only assume a set of *RGB* images *I* as inputs, with unknown camera parameters, and they seek to jointly optimize the camera parameters and scene representation during the training. Mathematically, this can be written as: $\Theta^{*},\Pi^{*}=\arg\min_{\Theta ,\Pi }\left[E\left(\hat{I},\Pi^{*}|I\right)\right]$.

**Figure 3:**
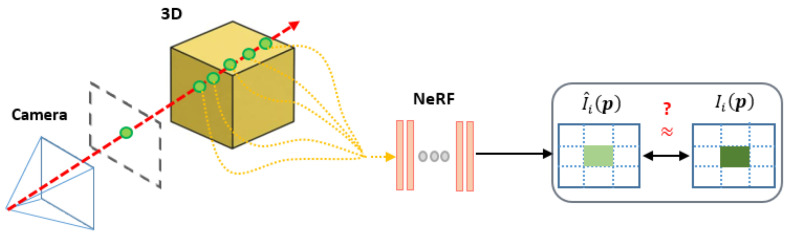
Summary of the NeRF-- framework. A NeRF model and the camera parameters of the input images are simultaneously optimized by minimizing the photometric reconstruction error ($E=\sum_{i=1}^{N}{∥I_{i}-{\hat{I}}_{i}∥}_{2}^{2}$) between the input and the reconstructed images, and a pixel, p is rendered once these camera parameters are optimized (read more details in the text body, and in [15]).

**Figure 4:**
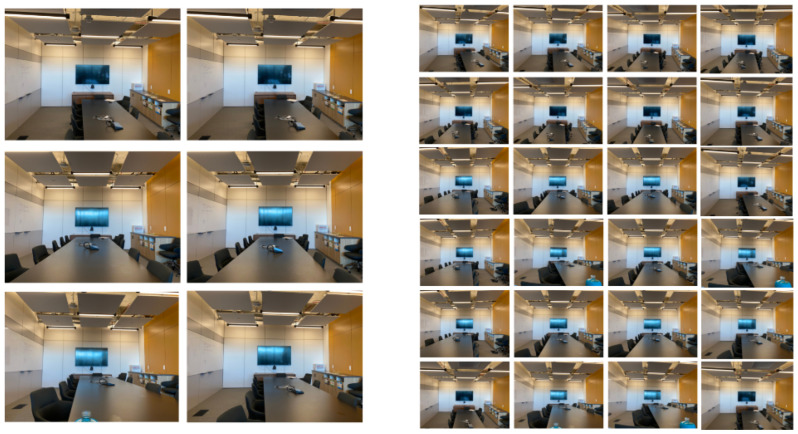
Image synthesis example. From a reduced number of input image views, NeRF-- renders a predefined number of high-quality new ones. Original input images can be downloaded from the corresponding repository in [30].

Figure 3 shows a brief scheme of the NeRF-- method (further graphical and mathematical details can be found in [15]). On the other hand, Figure 4 shows an example of the view synthesis results that the NeRF-- method obtains. Starting from a few images (six, in this case), NeRF-- generates synthesized views corresponding to other camera positions. As we can see, the results look realistic, with no color or geometrical artifacts whatsoever. Input views in Figure 4(left) were obtained from the web page created by the authors of the NeRF method [30].

We should emphasize that the above method (and potential variants that might appear in the future) could be good options as post-processing algorithms for Fourier lightfield data, mainly because they are able to reliably encode the scene information that is being acquired, and do not need other, more human-involved algorithms, such as in [31].

## 3. Results and Discussion

**Figure 5:**
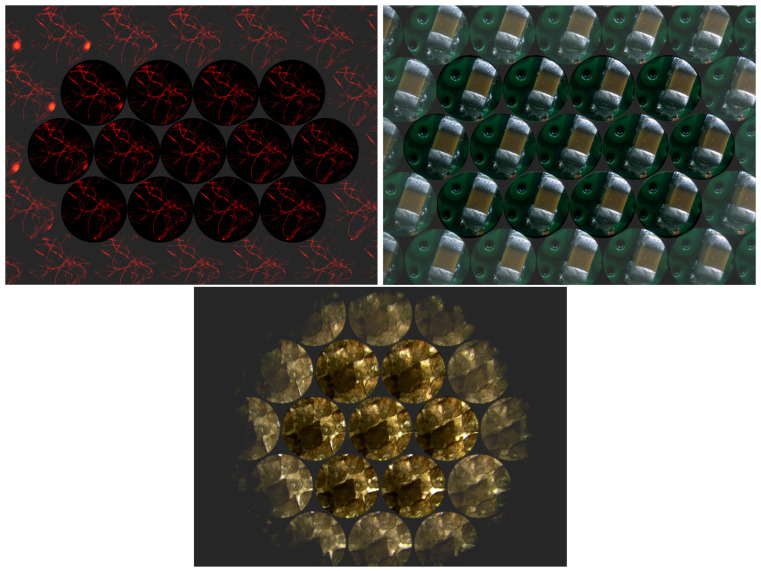
Integral image of the three types of scenes considered: *fiber*, *chip*, and *shark*. In the pictures, we have shaded the non-complete EIs.

A series of samples were prepared and subsequently acquired by an FLMic built in our laboratory in open configuration. In all the experiments, we used an MLA with pitch *p* = 1.0 mm and focal length *f*_L_ = 6.44 mm, and a sensor with 2560×1920 square pixels with size δ = 2.2 μm. Figure 5 shows representative examples of them. The first sample (top left) is composed of cotton fibers stained with the fluorescent ink of a highlighter. The second sample (top right) is the condenser of an electronic circuit. The third (bottom) corresponds to a dried shark skin tissue. We will call these samples *fiber*, *chip*, and *shark*, respectively, from now on.

Table 1 summarizes the main features of the FLMic setups used for the different acquisition sessions. The focal length of the second relay lens ($f_{R2}=100$ mm) is not specified in the table because it is the same for all the samples. Looking at both Figure 5 and Table 1, we can correlate the appearance of each sample with the illumination technique used to observe it. The *fiber* sample was imaged in epi-fluorescence mode; that is, it was illuminated through the objective by a monochromatic beam (λ=480 nm). The same objective collected the light emitted by the fluorescent fibers, producing an integral image in the sensor plane after blocking out the illumination beam by means of a dichroic mirror. The *chip* sample was epi-illuminated in reflection mode with white light. The background is the printed circuit board (PCB) of the electronic chip: since this sample is not transparent, it was observed with reflected light. Finally, the *shark* sample shows a yellowish background because it was trans-illuminated with white light.

Note that we have used different types of illumination techniques, aiming to test the performance of the algorithms for a diverse group of samples. The reader should also bear in mind that, in the integral images of *fiber* and *chip*, despite being *N_EI_* = 9, only five complete EIs can be seen in a row. This is because the exit pupil (the image of the AS through the relay system) was greater than the sensor we used. This fact has impacts on the lateral resolution of the captured EIs, but no impact on the performance of the view synthesis algorithm.

Considering only the complete EIs (i.e., those that can be seen entirely), the configuration of the integral image for the *fiber* and *chip* samples is four–five–four: four EIs in the first row, five in the second row, and four in the third row. In the integral image of *shark*, we have *N_EI_* = 4. As this is an even number, in the central row (corresponding to the diameter of the exit pupil), we have three complete EIs, and two half EIs. Therefore, the integral image configuration is two–three–two.

NeRF-- was applied, for each sample, using the acquired complete EIs as training images. The output is the synthesized images. Neural network convergence was analyzed based on the 2D inferred positions of the acquisition sensor. Those inferred positions accurately reproduce the hexagonal geometry of the MLA used for the image capture. The number of *epochs* considered was 500 for the *fiber*, and 1000 for the other two samples. Two hundred (200) new perspective images per scene were synthesized. To perform the tests, Google Colab was used, since it offers a greater computational power than our computers. The training time was 12 min for the *fiber*, 20 min for the *chip*, and 9 min for the *shark*. The test with the *chip* was repeated with a laptop with an Intel i7-6700HQ CPU and a Nvidia GTX 960M GPU. The training time in this case was 50 min.

**Figure 6:**
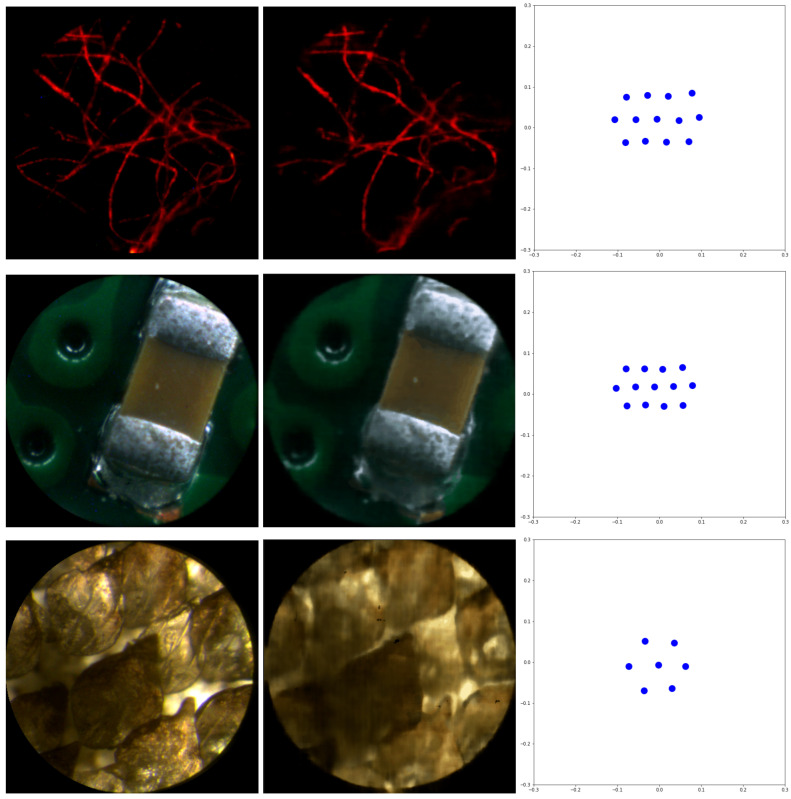
(**Left column**) Acquired elemental image; (**central column**) synthesized image; (**right column**) 2D inferred positions of the capturing cameras (blue full dots).

Figure 6 shows the following for the three selected samples: one of the captured EIs (left); one of the synthesized views (center). Comparison between the first and second columns shows us that the images synthesized by NeRF-- fully preserve the resolution in case of epi-illuminated sparse fluorescent samples, but suffer from a slight fall in resolution in the case of epi-illumination reflections in the brightfield samples. On the contrary, the resolution of the synthesized image in the case of the trans-illumination architecture is much poorer. The right-side column shows that the inferred capturing camera array reproduces the hexagonal structure of the physical MLA with good accuracy.

**Figure 7:**
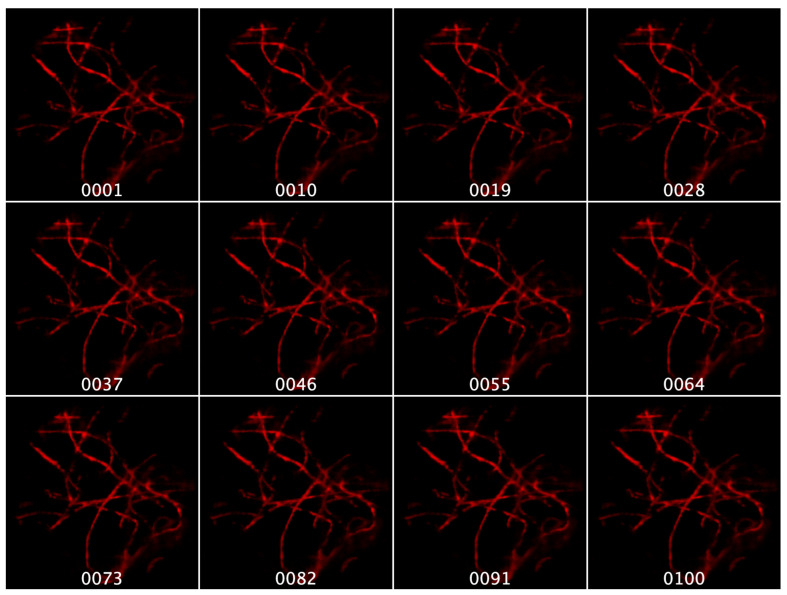
Group of 12 synthesized images extracted from the series of 200 images provided by NeRF-- for the case of the *fibers* sample. The labels refer to the position of each image in the order created during the generation of these images.

Next, in Figure 7, we show a series of images extracted from the 200 images on the *fiber* specimen generated by NeRF--. A thorough observation of the synthesized images demonstrates the homogeneous resolution of all the images, and the small changes of parallax between neighbor ones. Take into account that, in fact, the shown images are not neighbors, since there are still eight images between any two of those shown. The maximum parallax is achieved between images 1 and 100.

In order to gain a deeper insight into the utility of the proposed approach, it would be useful to generate some videos where the parallaxes of captured EIs, and the corresponding synthesized views, are compared. In this sense, we have prepared Video S1, which can be found in the Supplementary Material. In that video, we compare two movies. We show, on the left side, a movie whose frames are the captured EIs of the *fibers* sample, and on the right side, a movie with the 200 synthesized images. This is the best result achieved here. From the movie, we can confirm that all the synthesized images have been generated without any kind of resolution degradation. The perspective change is fairly smooth and continuous and, importantly, the occlusions are very well-resolved.

In Video S2, also given in the Supplementary Material, we show the movies corresponding to the *chip* sample. In this case, the synthesized views suffer from a slight lost in resolution, but the perspective change is very continuous and realistic. Finally, in Video S3, we show the movies corresponding to the *shark* sample. Clearly, this is the worst case in terms of resolution. However, it can still be of utility, since the synthesized perspective views are very realistic, and the occlusions are well resolved.

The reconstruction artifacts of Videos S2 and S3 might depend mostly on the nature of NeRF--. The estimation of the 3D model is difficult in images with low contrast and an absence of features, which are intrinsic characteristics of these microscopic images (mainly those of *shark*). In these conditions, the algorithm fails to predict a good 3D model of the sample, leading to artifacts in the reconstruction. This can be observed also in the original results presented by the authors of NeRF in [30]: in those regions of the scenes that present lack of features, defocus, or low contrast, the resolution of the synthesized images is substantially decreased. This effect seems to be more evident in images with a low number of pixels (455 × 455 pixels per EI). In addition, for the case of *shark*, the number of EIs is lower than that for the other two samples, which means that the model was trained with less images. This, along with the repetitive pattern and the typical background of brightfield images, might lead to a bad estimation of the 3D model, and hence to synthesized images with poorer resolutions and more artifacts.

Finally, to assess the view synthesis quality, we have made a new computer experiment in which, for the case of the *chip* sample, we have trained NeRF-- with all the EIs but the central one. The central EI is, therefore, used to assess the view synthesis quality. Then, we compared the EI acquired with the central microlens and the corresponding synthesized view in terms of the peak signal-to-noise ratio (*PSNR*). We obtained a value of PSNR=34.82, which is similar to the one obtained when the test image is not omitted in the training, and indicates a good image synthesis quality.

## 4. Conclusions

Fourier lightfield microscopy (FLMic) features a promising method for the fast capture of the plenoptic map of microscopic samples with high lateral resolution. The price paid is a low number of perspective images, which somehow compromises its utility in terms of display applications. This problem comes from the trade-off between the number of captured views and the effective numerical aperture (NA) value. Thus, a computational method that preserves the resolution but synthesizes new views is of great importance. In this sense, this paper presents, to the best of our knowledge, the first application of a neural radiance field-type method (NeRF--) for the synthesis of new views in FLMic. The application of the NeRF-- concept to FLMic is not trivial, due to some of its features, such as: (a) the telecentric nature of FLMic; (b) the object plane is acquired without any parallax, and the other planes show positive or negative parallax; (c) the particular type of illumination technique and the type of sample. In this sense, we found that, in the case of epi-illumination microscopy, the technique provides remarkable results, with only slight or no losses in lateral resolution, depending on the structural information of the images, but with continuous parallax and well-resolved occlusions. This can be of great utility for microscopists, since they can capture 3D dynamic samples in real time with the FLMic, and perform a post-processing application of NeRF-- for a realistic off-line observation of the sample from a continuous viewpoint.

Future work will be focused on the development of a new mathematical framework that is able to take into account the particular details of the Fourier lightfield microscope used, in order to improve the quality of the reconstruction.

## Figures and Tables

**Table 1:** Acquisition data for the different samples. First column: the microscope objective information is shown in the classical magnification/numerical aperture format. Second column: R1 lens focal length. Third column: number of EIs. Fourth column: illumination technique.

Sample	MO	*f*_R1_	*N_EI_*	Illumination Type
*fiber*	10×/0.45	200 mm	9	*Epi-Fluorescence*
*chip*	10×/0.45	200 mm	9	*Epi-Reflection*
*shark*	20×/0.40	180 mm	4	*Brightfield*

## Data Availability

Data is available upon request to the authors on a reasonable basis.

## Supplementary Materials

The following supporting information can be downloaded at: https://www.mdpi.com/article/10.3390/s22093487/s1, Video S1: Animation showing the *fiber* sample from the inferred and synthesized views; Video S2: Animation showing the *chip* sample from the inferred and synthesized views; Video S3: Animation showing the *shark* sample from the inferred and synthesized views.

## Abbreviations

The following abbreviations are used in this manuscript:

3D	Three-dimensional
AS	Aperture stop
EI	Elemental image
FLMic	Fourier lightfield microscopy
IEP	Inferred extrinsic parameters
MLA	Microlens array
MLP	Multilayer perceptron
NeRF	Neural radiance field
NA	Numerical aperture
NVS	Novel view synthesis
PCB	Printed circuit board
R	Relay
SfM	Structure-from-motion
VR	Virtual reality
